# Supplementary material for: Blood flow restriction reduces the increases in cardiorespiratory responses and subjective burden without inhibiting muscular activity during cycling at ventilatory threshold in healthy males
Source: PLoS One. 2023 Dec 8;18(12):e0294524. doi: 10.1371/journal.pone.0294524 (PMC10707540; doi:10.1371/journal.pone.0294524)
Supplement: Attachment — Submitted filename: ResponseToReviewer2.docx [file pone.0294524.s001.docx]

*Major comments:*

*It is this reviewer’s opinion that the results do not necessarily support the authors conclusion. A standard ramp protocol that increases intensity every minute was used to determine VT. This was performed with and without blood flow restriction. However, this type of protocol does not result in steady state cardiovascular responses that are typically observed with longer stages. Thus we still do not know if 5, 10, 15 minutes of cycling at VT with KAATSU will result in lower cardiorespiratory responses compared to VT without KAATSU. This may be particularly true with KAATSU. It’s possible that the build up of metabolic by products would result in a greater exercise pressor reflex with time leading to a continued rise in the cardiorespiratory response during steady state cycling with KAATSU that would not be observed while cycling without KAATSU.*

**Response:** Thank you for raising this important point. We have added further study limitation and future experiment plan based on your suggestion. Page 9, lines 299-330

*There is no explanation on why VT occurs at a lower power output with KAATSU compared to without KAATSU. Why would Ve/VO2 increase earlier (at a lower power output and lower VO2) with KAATSU compared to without KAATSU. Does VT have the same physiological mechanism and meaning during KAATSU cycling compared to traditional cycling.*

**Response:** We have added potential explanations in the Discussion section. Page 8, lines 252-257

*Most other literature indicates that EMG activity does increase with KAATSU. Why does your data not agree with previous reports?*

**Response:** We agree that most previous studies report increases in EMG activity during KAATSU vs. no-KAATSU exercise at the same exercise intensity. In this study, we compared data based on cardiorespiratory responses i.e., at VT. In this comparison, EMG activity was similar between KAATSU vs. no-KAATSU. Thus, when the comparison is done at VT, EMG activity under the two conditions are not different.

*This manuscript would read much better if the authors minimized the use of the phrases “KAATSU at VT” and “no-KAATSU at VT”. If the authors would more clearly state that all data used in the analysis were data that occurred at VT (this is not mentioned in the data analysis section), then the authors would not have to continuously state “at VT” throughout the entire manuscript. The readers should be reminded in the discussion that the data occurred at VT, but the constant use of “at VT” is overwhelming and makes it difficult to read.*

**Response:** Thank you for your kind suggestion. We have reduced “at VT” from the main text as much as we can to increase readability.

*In addition, my above point also is relevant to the figures. The y axis should be HR at VT and VO2 at VT while the x-axis are simply KAATSU and no-KAATSU.*

**Response:** We have revised x- and y-axis name in Fig 1-3.

*There should be some discussion regarding the fact that individuals with cardiovascular disease tend to have greater exercise pressor response which could lead to elevated blood pressure and greater risks.*

**Response:** We have added this limitation and future study plan in the limitation paragraph at the end of Discussion section. Page 9, lines 299-330

*Minor Comments*

*With regards to determination of VT, were the methods consistent between visits for each subject. If the VT for one subject was determined by V-slope method on the first visit, was the V-slope method automatically used for the second visit? What if VT was calculated by ventilatory equivalents on the first visit, but that method could not be used for the second visit?*

**Response:** The method for determining VT was same with in each participant.

*EMG was measured across 5 cycles. Were these 5 consecutive cycles or simply 5 cycles randomly selected at VT? Why was EMG data presented at mean(SD) while all other data was presented as mean (SE)?*

**Response:** Peak and averaged amplitude for knee joint extension phase of a series of 5 cycles within the range of 1 min before VT were computed. According to your suggestion, EMG data was also presented as mean±SD. We have revised the results of EMG activity. Page 6, lines 181-182, page 8, lines 235-247, and Fig 3

*Why does figure 1 have **? This is not explained in the figure legend.*

**Response:** We have revised the legend of Fig 1. Page 6, lines 203-204

*Line 133-135: please clarify. loss of muscle function…. Are you referring to fatigue?*

**Response:** We have revised these sentences. Page 4, lines 131-134

*Line 283-285: Cardiorespiratory responses were reduced with KAATSU likely due to reduced power at VT. Training at this lower cardiovascular level would likely lead to reduced cardiovascular adaptations compared to those that might be achieved with higher cardiovascular stress non-KAATSU cycling at VT.*

**Response:** We partially agree with your comment, but suggest that KAATSU aerobic exercise at VT will greatly benefit low fit patients and those who are frail and participate in cardiac rehabilitation. Page 9 lines 292-297.

*Line 278-279: This statement has very little meaning unless you include the fact that the studies you are referencing (10,11) used KAATSU.*

**Response:** We have revised this sentence. Page 9, lines 279-280
