# Supplementary material for: Blood flow restriction reduces the increases in cardiorespiratory responses and subjective burden without inhibiting muscular activity during cycling at ventilatory threshold in healthy males
Source: PLoS One. 2023 Dec 8;18(12):e0294524. doi: 10.1371/journal.pone.0294524 (PMC10707540; doi:10.1371/journal.pone.0294524)
Supplement: Attachment — Submitted filename: ResponseToReviewer2.docx [file pone.0294524.s002.docx]

Thank you for the careful review of the manuscript.

*Abstract*

*I don't feel that the last statement in the abstract is necessary.*

**Response:** We have removed the last statement from the Abstract.

*Line 181 should read, ".... compared between KAATSU and no-KAATSU.."*

**Response:** We have removed “with” from the sentence. Page6, lines 180-181

*Line 186-190: when you are referring to "peak" are you referring to "peak power output"? If so please indicate that.*

**Response:** We have revised sentences including “peak” throughout the manuscript.

*Line 264-265 should read, "...and cardiac work at VT indexed by DP was significantly lower with KAATSU compared to no-KAATSU."*

**Response:** According to your comment, we have removed “at VT” from “KAATSU at VT” and “no-KAATSU at VT” throughout the manuscript.

*Thank you for including the limitation with the VT protocol. However, I think it could be more clearly stated as*

*"Third, our examination of cardiorespiratory and subjective responses during KAATSU aerobic exercise at VT was a single point from a one-minute stage of a ramp protocol. It is likely that the cardio-respiratory response at each stage did not achieve steady state and may not reflect the responses of more prolonged exercise (ie 10-30 minutes) at that same intensity."*

**Response:** We agree and have revised this text. Page 10 lines 310-314
